# Assessment of salicylic acid and potassium nitrate to mitigate frost stress in autumn-sown potato crop cv. Sutlej

**DOI:** 10.1038/s41598-025-85769-7

**Published:** 2025-01-14

**Authors:** Muhammad Wasim Haider, Syed Mohsin Abbas, Tanveer Hussain, Muhammad Tahir Akram, Umar Farooq, Mona S. Alwahibi, Mohamed S. Elshikh, Zaid Shakeel, Muhammad Nafees, Muhammad Rizwan, Rashid Iqbal

**Affiliations:** 1 Department of Horticultural Sciences, Faculty of Agriculture and Environment, The Islamia University of Bahawalpur, Bahawalpur, 63100 Pakistan; 2 Department of Horticulture, Faculty of Agricultural Sciences, University of the Punjab Lahore, Lahore, 54590 Pakistan; 3 Department of Horticulture, Faculty of Agriculture, PMAS-Arid Agriculture University, Rawalpindi, 46300 Pakistan; 4 Department of Botany and Microbiology, College of Science, King Saud University, Riyadh, 11451 Saudi Arabia; 5 Institute of Crop Science and Resource Conservation (INRES), University of Bonn, 53115 Bonn, Germany; 6 Department of Agronomy, Faculty of Agriculture and Environment, The Islamia University of Bahawalpur, Bahawalpur, 63100 Pakistan; 7 Department of Life Sciences, Western Caspian University, Baku, Azerbaijan

**Keywords:** Antioxidative enzymes, Fluorescence-related metrics, Frost stress mitigator, Potassium nitrate, Proline, Reactive oxygen species, Salicylic acid, Tuber yield., Plant sciences, Environmental sciences

## Abstract

Potato is cultivated all the year round in Pakistan. However, the major crop is the autumn crop which is planted in mid-October and contributes 80–85% of the total production. The abrupt climate change has affected the weather patterns all over the world, resulting in the reduction of the mean air temperature in autumn by almost 1.6 °C in Pakistan, which in turn, has adversely affected the crop performance and tuber yield. This trial, therefore, was conducted to optimize and evaluate the concentration of salicylic acid (SA) and potassium nitrate (KNO_3_) for inducing frost stress tolerance in an autumn-sown potato crop cv. Sutlej. The findings revealed that the foliar application of 0.5 mM SA significantly enhanced the growth, yield, fluorescent, and biochemical indices of potato plants outperforming 100 mM KNO₃ application in comparison with the control. This included increased plant height by 14% and 17.6%, leaf area index by 6.3% and 26.3%, shoot biomass by 15.4% and 46.2%, crop growth rate by 16.7% and 43.3%, average tuber weight by 8.2% and 23%, tuber diameter by 6.8% and 12.2%, tuber yield by 26.1% and 46.3%, leaf angle by 16.2% and 21.6%, quantum yield of photosystem II by 20.6% and 28.2%, photosynthetically active radiations by 20.5% and 32.4%, chlorophyll content by 6.3% and 14.6%, leaf thickness by 14% and 29%, linear electron flow by 20% and 32.7%, O^–2^ by 6% and 14.4%, H_2_O_2_ by 11.7% and 27.6%, enzyme activities of catalase by 20.7% and 28.5%, superoxide dismutase by 28.6% and 28.5%, peroxidase by 8.3% and 13.5%, ascorbate peroxidase by 17.2% and 37.8%, total protein by 21% and 37%, proline by 36.2% and 114%, and phenolic content by 33% and 63.3% with a reduction in non-photochemical quenching by 12.7% and 29.6%, non-regulatory energy dissipation by 169.5% and 268.5%, and leaf electrolyte leakage by 57.5% and 180%, compared to KNO_3_ and the control, respectively. Based on the above findings, it can be concluded and recommended that 0.5 mM foliar spray of SA can be utilized on potato crop cv. Sutlej in frost-sensitive regions. However, the application rate of KNO_3_ needs to be optimized in order to use its maximal frost stress tolerance potential.

## Introduction

Potato is one of the most consumed food crops worldwide, playing a key role in food security and economic stability particularly in developing countries like Pakistan where it has a high share in the rural livelihood^1^. Potato is cultivated on an area of 0.31 million hectares in Pakistan, with an estimated yearly production of 7.93 million tons, on an average of 25.3 tons ha^–1^^2^. There is often a gap between current and potential crop yields^3^. The “yield potential concept” highlights that biotic and/or abiotic stressors might hinder potato crop growth and tuber development in natural production systems, preventing full yield^1,4,5^.

Plant stress is a condition of plant growth under unfavorable circumstances^6^. The impact of stress can lead to growth deficiency, yield drop, and permanent damage or even death if the stress exceeds the plant tolerance limits^7^. Abrupt climate change is a great challenge for researchers in sustainable crop production^8^. This has changed the patterns of weather, resulting in extremes of drought, heat, and frost^9^. The negative impact of abiotic stresses on plant performance and crop yield is likely to be increased in the future due to continued greenhouse gas emissions^10^.

Frost damage takes place when the ground surface temperature fluctuates between cold and warm states, with thermal characteristics and freezing frequency^11^. Frost injury symptoms include tissue browning, blackening, and wilting of leaves and stems^12^. Potatoes are sensitive to the frost damage, particularly during emergence, sprout development, and tuber initiation^13^. Frost tolerance is often judged from the extent of deterioration of morphological indices^14^.

The key physiological processes disturbed by the onset of frost include the synthesis of glucose in the source leaves via photosynthesis, the translocation of the end product of photosynthesis, sucrose, to the stolon; and the conversion of sucrose to starch in the stolon for the initiation and growth of potato tubers^10^. Frost is assumed to impair these physiological and biochemical components of plant growth^13,15^. The coordination of these processes defines tuber production and quality^16^.

The application of frost mitigating agents is essential to protect potato crop from frost stress^17^. Several chemicals like salicylic acid and potassium nitrate are reported in various crops against various stresses to offer a cost-effective solution for extending the growing season and lowering the crop losses^18–23^. They provide a critical protection against unpredictable frost, ensuring sustainable production.

Salicylic acid (SA), orthohydroxy benzoic acid, belongs to phenolic phytohormones that regulate plant metabolism and signal molecules necessary for the expression of the genes involved in plant defense reactions^24^. It improves photosynthesis and antioxidative protection in plants^25^. Previously, endogenous SA activated the processes responsible for the improvement of cold resistance in cucumber plants^26^. In another study, SA substantially improved the low temperature stress tolerance through improving growth and accumulation of antioxidative enzymes activities and osmoprotectants^27^. The foliar spray of SA elevated the rate of photosynthesis, efficiency of photosystem II, chlorophyll content, and seed yield in Brassica juncea^28^. SA has also been found to improve the growth, photosynthetic efficiency, and quality of the pea^29^.

Foliar application of potassium plays an important role in balancing membrane potential and turgor^30^. It activates antioxidative enzymes, regulates osmotic pressure, stomatal opening, and membrane integrity^31^. Previous studies revealed that supplying low levels of KNO_3_ could alleviate the NaCl-induced stress in certain grass species^32^. In another study, potato plants sprayed with potassium salts had the highest tuber yield and quality compared to the control plants^33^. Similarly, in another investigation, an increase in the tuber yield to potassium nitrate has been observed in a trial carried out by Wibowo et al.^34^. Furthermore, the crop growth and tuber yield of potato plants significantly improved with the application of potassium silicate^35^.

The above literature indicates the role of both SA and KNO_3_ in mitigating abiotic stresses. However, how these chemicals improve frost stress tolerance by enhancing photosynthetic responses of potato plants is not yet fully understood. Therefore, this study was set out to compare the effect of SA and KNO_3_ on growth, photosynthesis, yield, and quality related indices of potato cv. Sutlej under frost stress.

## Results

### Trend of air temperature at the experimental site

**Fig. 1:**
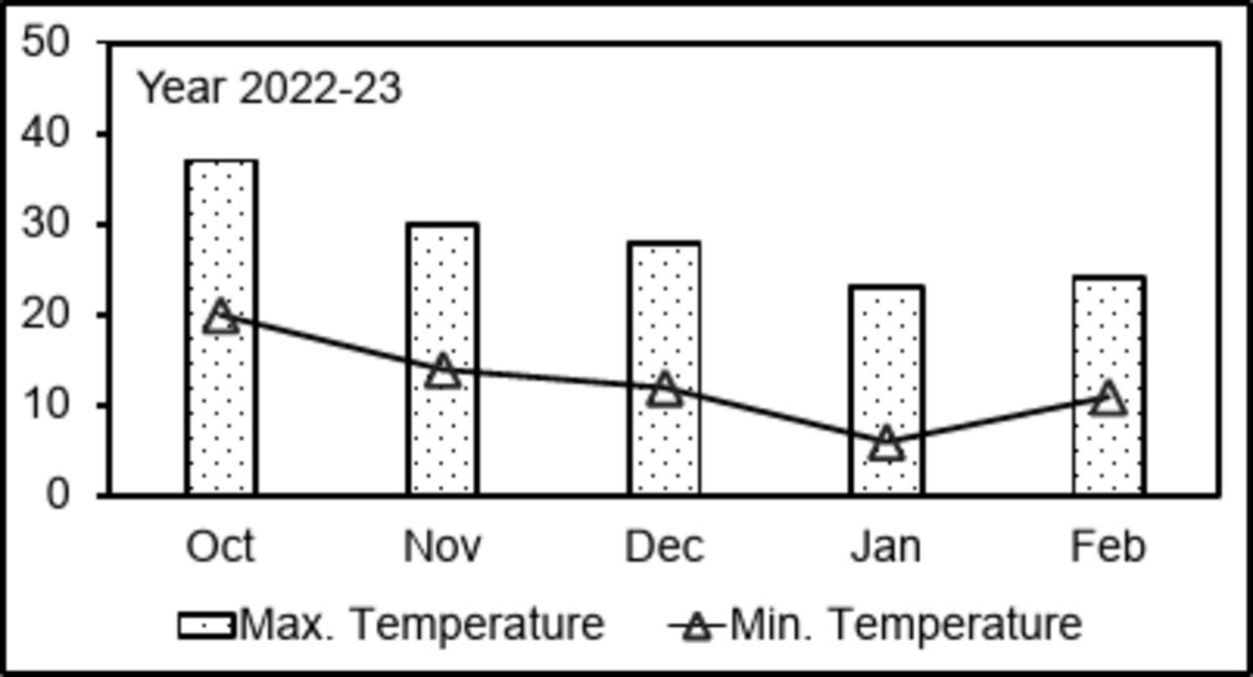
Maximum and minimum air temperatures recorded for the study area during the experimental duration in 2022–23.

The maximum air temperature showed an erratic drop from the first month (October) (37 °C) of the experimental duration until the second-last month (January) (23 °C) (Fig. 1). Then, it slightly increased in the last month (February) (24 °C) of the experimental duration (Fig. 1). The minimum air temperature also showed a declining trend from the October (20 °C) to the January (6 °C) and then suddenly rose in the February (11 °C) (Fig. 1). However, it showed a highest drop in the month of January by approximately 14 degrees leaving potato crop at a frost risk (Fig. 1).

### Physico-chemical examination of soil and irrigated water

The selected physico-chemical characteristics of the top 0–15 and 16–30 cm soil are presented separately in Table 1. The soil was moderately alkaline with pH ranging between 8.1 and 8.4 and EC ranging between 1.65 and 1.82 dS m^–1^ (Table 1). The clay constituents in the soil ranged between 14 and 18%, silt between 25 and 27%, and sand between 57 and 59%. Thus, the textural class was sandy loam (Table 1). The soil’s cation exchange capacity (CEC) ranged between 7.6 and 8.0 c mol kg^–1^ (Table 1). This could be attributed to the build-up of a high concentration of salts (0.70–0.95 g Kg^–1^) in the soil of the experimental site (Table 1). The content of organic matter in the soil ranged between 0.82 and 0.88% (Table 1). The concentration of total nitrogen ranged between 0.043 and 0.056% (Table 1). The available P and K concentrations ranged between 10.5 and 13.2 mg kg^–1^ and 125–156 mg kg^–1^, respectively (Table 1). The measured physico-chemical characteristics of the irrigated water are presented in Table 2. The irrigated water also had a medium alkalinity and sodicity risk (Table 2).

**Table 1:** Physico-chemical examination of the soil at the study site.

Particular	Values obtained for both soil layers		Unit
	0–15 cm	16–30 cm	
Clay	18	14	%
Silt	25	27	%
Sand	57	59	%
Soil texture	Sandy loam	Sandy loam	-
Saturation	35	33	%
EC	1.82	1.65	dS m^–1^
pH	8.1	8.4	–
Total dissolved salts	0.95	0.70	g Kg^–1^
CEC	8.0	7.6	c mol kg^–1^
Organic matter	0.88	0.82	%
Total N	0.056	0.043	%
Available P	10.5	13.2	mg kg^–1^
Available K	156	125	mg kg^–1^

**Table 2:** Physico-chemical examination of the water irrigated at the study site.

Particular	Value	Unit
Total dissolved salts	1.24	g L^–1^
Ca^+ 2^ + Mg^+ 2^	7.35	Meq L^–1^
Na + 1	4.91	Meq L^–1^
HCO_3_^–1^	2.63	Meq L^–1^
Cl^–1^	1.10	Meq L^–1^
CO_3_^–2^	-	Meq L^–1^
EC	1.26	dS m^–1^
pH	7.9	–
Sodium adsorption ratio	2.50	–
Residual sodium carbonate	0.32	Meq L^–1^

### Morphological indices in potato cv. Sutlej

**Fig. 2:**
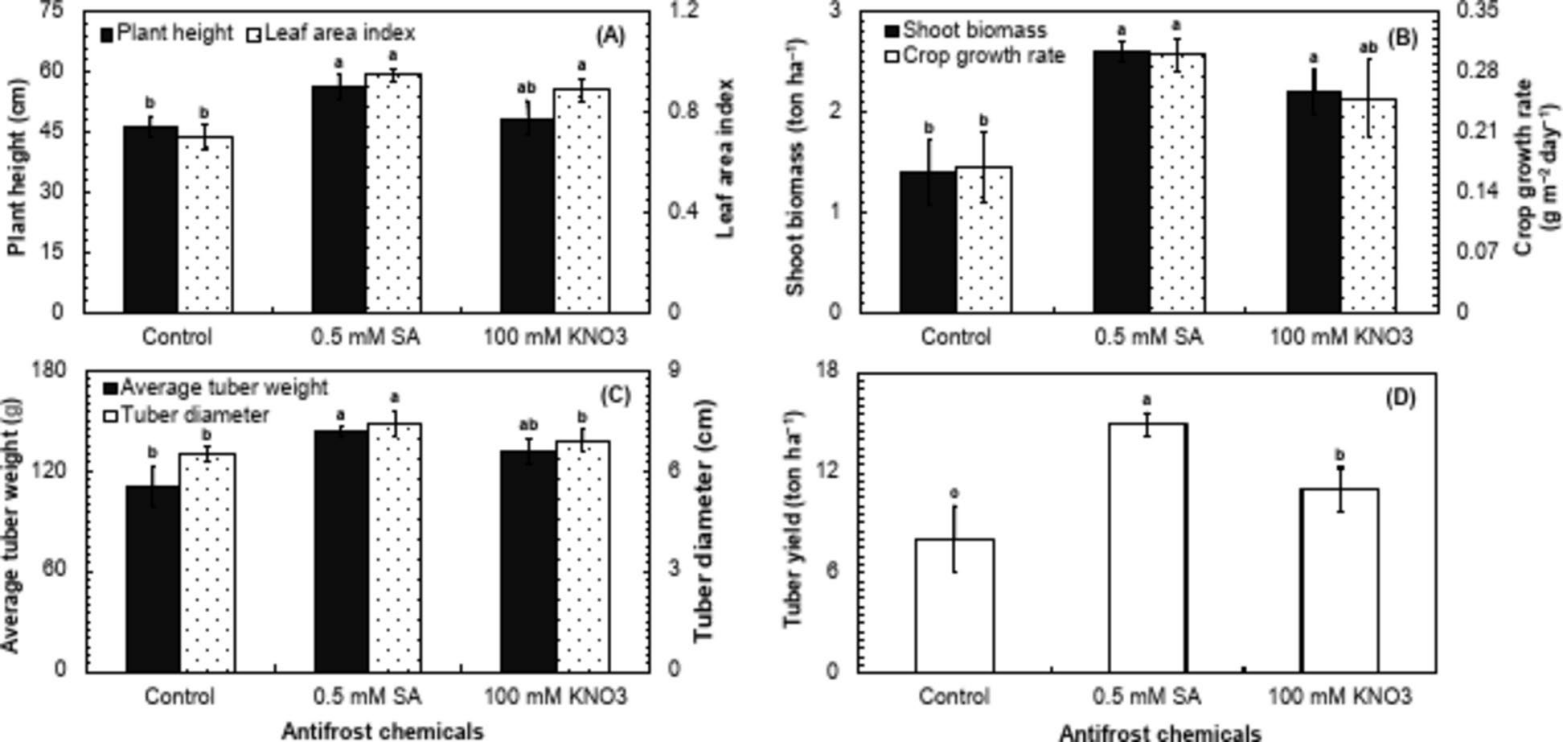
Comparison of the impact of salicylic acid (SA), potassium nitrate (KNO_3_), and a control for plant height and leaf area index (**A**), shoot biomass and crop growth rate (**B**), average tuber weight and tuber diameter (**C**), and tuber yield (**D**) in potato cv. Sutlej. The bars represent the standard error (±) of the mean (*n* = 4). Lettering indicates the differences in the treatment means that was conducted through the least significant difference (LSD) test at *P* ≤ 0.05 after analysis of variance.

The morphological indices are crucial to assess the efficacy of salicylic acid (SA) and potassium nitrate (KNO_3_) in increasing the frost stress tolerance and overall performance of autumn-sown potato crop. In this study, the foliar applications of SA and KNO_3_ were found to have a significant (*P* ≤ 0.05) effect on plant height, leaf area index (LAI), crop growth rate (CGR), and average tuber weight, in comparison with control (Table 3). The highly significant (*P* ≤ 0.01) variations were observed for the shoot biomass, tuber diameter, and tuber yield of potato cv. Sutlej in response to the foliar applications of SA and KNO_3_ when compared with the control (Table 3). The foliar application of 0.5 mM SA improved plant height and LAI of potato plants by about 14% and 6.3%, respectively than 100 mM KNO_3_, whereas, around 17.6% and 26.3% than the control (Fig. 2A). Furthermore, the application of 0.5 mM SA on potato plants also improved the shoot biomass and CGR by 15.4% and 16.7%, correspondingly than the KNO_3_, and 46.2% and 43.3% than the control (Fig. 2B). The average tuber weight and tuber diameter were found to be about 8.2% and 6.8% higher in the potato plants received foliar treatment in the form of 0.5 mM SA, compared to the KNO_3_ and about 23% and 12.2% higher compared to the control (Fig. 2C). In the end, the tuber yield was about 26.1% higher in the plants sprayed with 0.5 mM SA compared to the KNO_3_ and 46.3% higher compared to the control (Fig. 2D).

**Table 3:** Analysis of variance for morphological indices including plant height (PH), leaf area index (LAI), shoot biomass (SB), crop growth rate (CGR), average tuber weight (ATW), tuber diameter (TD), and tuber yield (TY) in the field-grown potato cv. Sutlej sprayed with two different antifrost chemicals (0.5 mM salicylic acid and 100 mM potassium nitrate) in comparison to the control.

Source of variance	PH	LAI	SB	CGR	ATW	TD	TY
Chemicals	90.05*	93.72*	94.56**	80.78*	93.30*	98.46**	97.55**
Error	9.85	6.25	3.50	11.27	6.62	1.53	2.45

*Significant at *P* ≤ 0.05.

**Significant at *P* ≤ 0.01.

### Fluorescent indices in potato cv. Sutlej

**Fig. 3:**
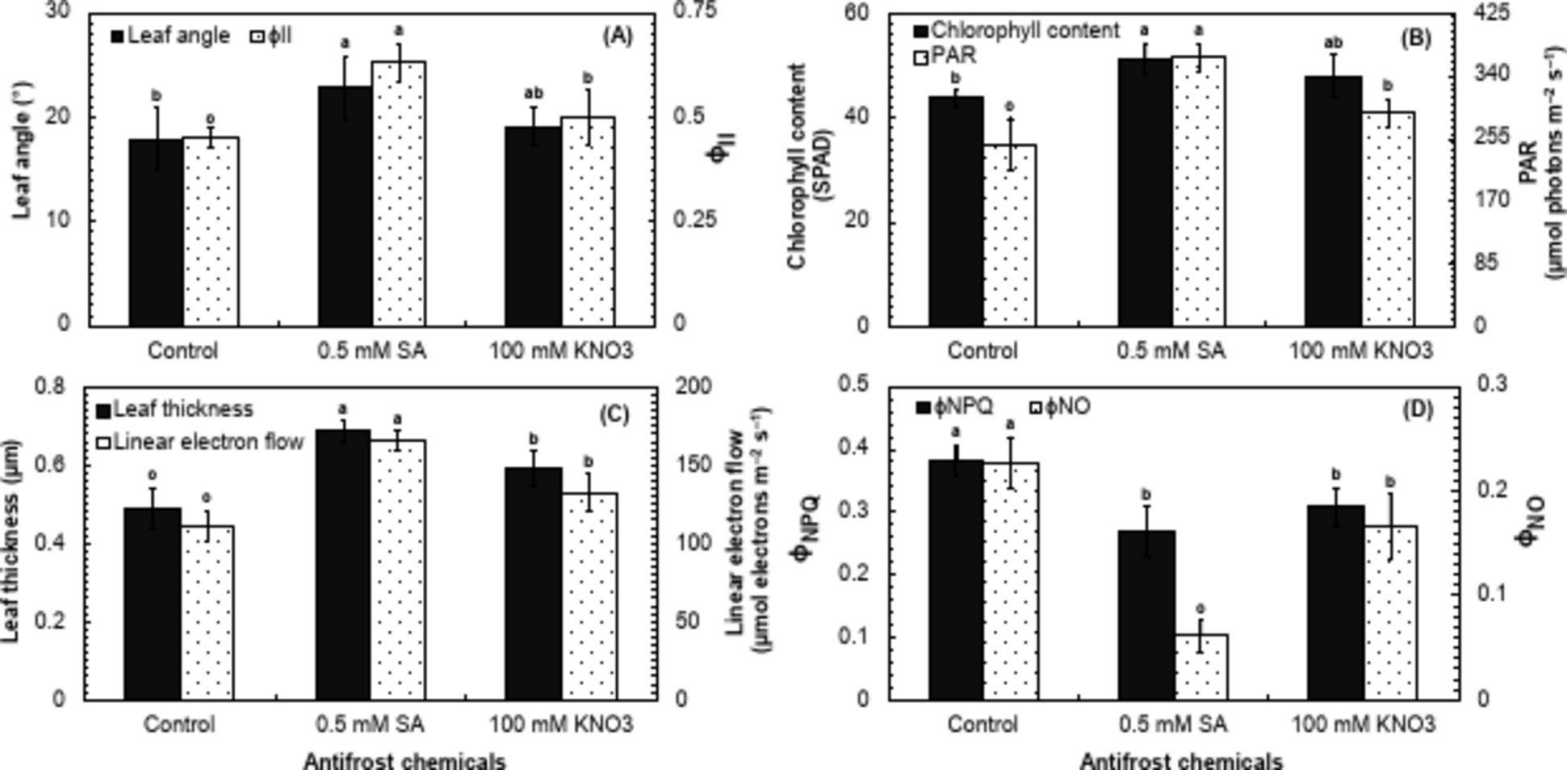
Comparison of the impact of salicylic acid (SA), potassium nitrate (KNO_3_), and a control for leaf angle and quantum yield of photosystem II (ΦII) (**A**), chlorophyll content and photosynthetically active radiation (PAR) (**B**), non-photochemical quenching (ϕ_NPQ_) and non-regulatory energy dissipation (ϕ_NO_) (**C**), and leaf thickness and linear electron flow (**D**) in the leaves of potato cv. Sutlej. The bars represent the standard error (±) of the mean (*n* = 4). Lettering indicates the differences in the treatment means that was conducted through the least significant difference (LSD) test at *P* ≤ 0.05 after analysis of variance.

The fluorescence-related indices are essential to evaluate the effects of salicylic acid and potassium nitrate on the efficiency of photosynthetic apparatus and frost tolerance of autumn-sown potato plants. In the current study, all the fluorescent attributes excluding leaf angle, leaf thickness, and linear electron flow were found to have highly significant (*P* ≤ 0.01) variations under the influence of SA and KNO_3_ in comparison with the control (Table 4). The leaf angle was found to have a significant difference (*P* ≤ 0.05) under the effect of SA and KNO_3_ in comparison with the control (Table 4). The foliar application of 0.5 mM SA increased the angle of leaves and the quantum yield of photosystem II (Φ_II_) by 16.2% and 20.6%, respectively compared to the 100 mM KNO_3_ application, and about 21.6% and 28.6% than the control (Fig. 3A). The fraction of the photosynthetically active radiations (PARs) and chlorophyll content were also enhanced by about 20.5% and 6.3% with the application of 0.5 mM SA than the KNO_3_ and by 32.4% and 14.6% than the control (Fig. 3B). Moreover, the leaf thickness and linear electron flow (LEF) were about 14% and 20% higher in potato plants sprayed with 0.5 mM SA than 100 mM KNO_3_ and 29% and 32.7% higher than the control (Fig. 3C). On the other hand, the processes of non-photochemical quenching (Φ_NPQ_) and non-regulatory energy dissipation (Φ_NPQ_) were found to be about 12.7% and 169.5% lower in the plants receiving foliar spray of 0.5 mM SA than the KNO_3_ and 29.6% and 268.5% lower than the control (Fig. 3D).

**Table 4:** Analysis of variance for leaf angle (LA), quantum yield o photosystem II (Φ_II_), chlorophyll content, photosynthetically active radiation (PAR), leaf thickness (LT), linear electron flow (LEF), non-photochemical quenching (ϕ_NPQ_), and non-regulatory energy dissipation (ϕ_NO_) in the leaves of the field-grown potato cv. Sutlej sprayed with two different antifrost chemicals (0.5 mM salicylic acid and 100 mM potassium nitrate) in comparison to the control.

Source of variance	LA	Φ_II_	Chlorophyll	PAR	LT	LEF	Φ_NPQ_	Φ_NO_
Chemicals	93.30*	98.46**	96.40**	98.49**	82.53*	85.31*	97.55**	98.83**
Error	6.62	1.53	3.55	0.19	9.80	7.94	2.45	1.15

*Significant at *P* ≤ 0.05.

**Significant at *P* ≤ 0.01.

### Biochemical indices in potato cv. Sutlej

**Fig. 4:**
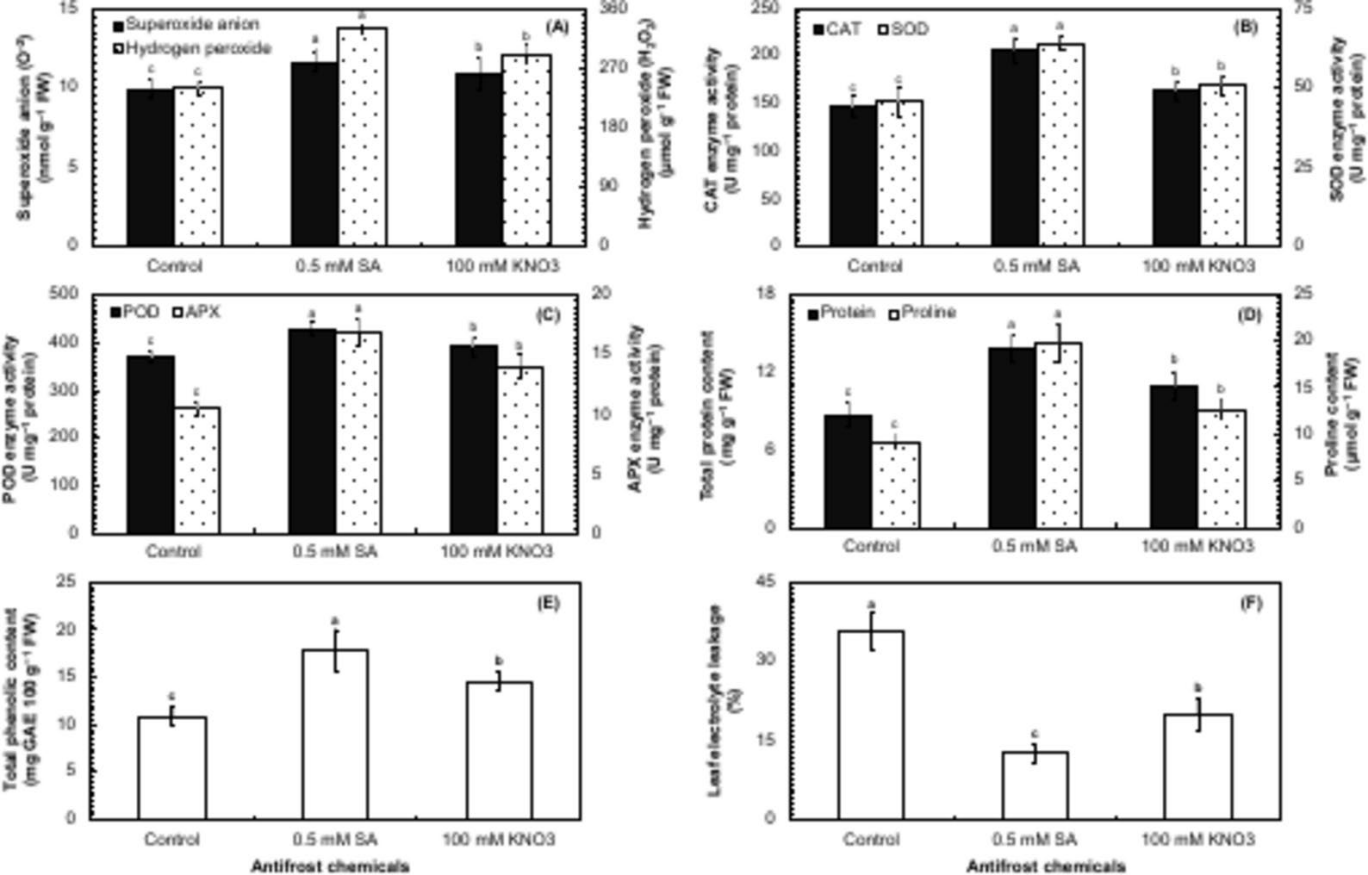
Comparison of the impact of salicylic acid (SA), potassium nitrate (KNO_3_), and a control for superoxide anion (O–2) and hydrogen peroxide (H_2_O_2_) (**A**), catalase (CAT) and superoxide dismutase (SOD) enzyme activities (**B**), peroxidase (POD) and ascorbate peroxidase (APX) enzyme activities (**C**), total protein and proline content (**D**), electrolyte leakage (**E**), and total phenolic content (**F**) in the leaves of potato cv. Sutlej. The bars represent the standard error (±) of the mean (*n* = 4). Lettering indicates the differences in the treatment means that was conducted through the least significant difference (LSD) test at *P* ≤ 0.05 after analysis of variance.

The biochemical indices like production of reactive oxygen species (superoxide anion and hydrogen peroxide) and antioxidative enzyme activities (catalase, superoxide dismutase, peroxidase, and ascorbate peroxidase) as a result of oxidative stress as well as the levels of proteins, phenolics and osmolytes (proline) are important to determine the endogenous metabolic changes occurring in response to foliar spray of salicylic acid and potassium nitrate on the autumn-sown potato plants under frost stress. In the present study, all the studied biochemical indices of potato cv. Sutlej excluding superoxide anion (O^–2^) and phenolic content were found to have highly significant (*P* ≤ 0.01) variations under the impact of anti-frost chemicals compared to the control (Table 5). The application of salicylic acid and KNO_3_ had a significant (*P* ≤ 0.05) effect on O^–2^ and phenolics (Table 5). The accumulation of O^–2^ and H_2_O_2_ content was about 6% and 11.7% higher in the leaves of potato plants sprayed with 0.5 mM SA, respectively than 100 mM KNO_3_ and 14.4% and 27.6% higher than the control (Fig. 4A). As a result, the enzyme activities of catalase (CAT) and superoxide dismutase (SOD) were about 20.7% and 28.6% higher in the plants receiving foliar application of 0.5 mM SA, respectively compared to 100 mM KNO_3_ and 28.5% higher compared to the control (Fig. 4B). Furthermore, the application of 0.5 mM SA also improved the enzyme activities of peroxidase (POD) and ascorbate peroxidase (APX) in the leaves of potato plants by almost 8.3% and 17.2%, respectively compared to 100 mM KNO_3_ and about 13.5% and 37.8% compared to the control (Fig. 4C). The total protein content was also increased by 21% in the leaves with the application of 0.5 mM SA on potato plants compared to 100 mM KNO_3_ and about 37% compared to the control (Fig. 4D). The proline content was found to be 36.2% higher in the potato plants sprayed with 0.5 mM SA than 100 mM KNO_3_, and about 114% higher compared to the control (Fig. 4D). The phenolic content in the leaves of potato plants was found to increase by around 33% with the application of 0.5 mM SA than 100 mM KNO_3_ and about 63.3% than the control (Fig. 4E). The electrolyte leakage in the leaves of potato plants was decreased by around 57.5% with the application of 0.5 mM SA compared to 100 mM KNO_3_ and about 180% compared to the control (Fig. 4F).

**Table 5:** Analysis of variance for superoxide anion (O^–2^), hydrogen peroxide (H_2_O_2_), catalase (CAT), superoxide dismutase (SOD), ascorbate peroxidase (APX), total protein content, proline concentration, leaf electrolyte leakage, and total phenolic content in the leaves of field-grown potato cv. Sutlej sprayed with two different antifrost chemicals in comparison to the control.

Source of variance	O^–2^	H_2_O_2_	CAT	SOD	POD	APX	Protein	Proline	Phenolics	LEL
Chemicals	93.30*	98.46**	96.40**	98.49**	97.55**	98.83**	97.51**	99.63**	86.97*	97.88**
Error	6.62	1.53	3.55	0.19	2.45	1.15	2.43	0.14	10.48	1.88

*Significant at *P* ≤ 0.05.

**Significant at *P* ≤ 0.01.

## Discussion

**Fig. 5:**
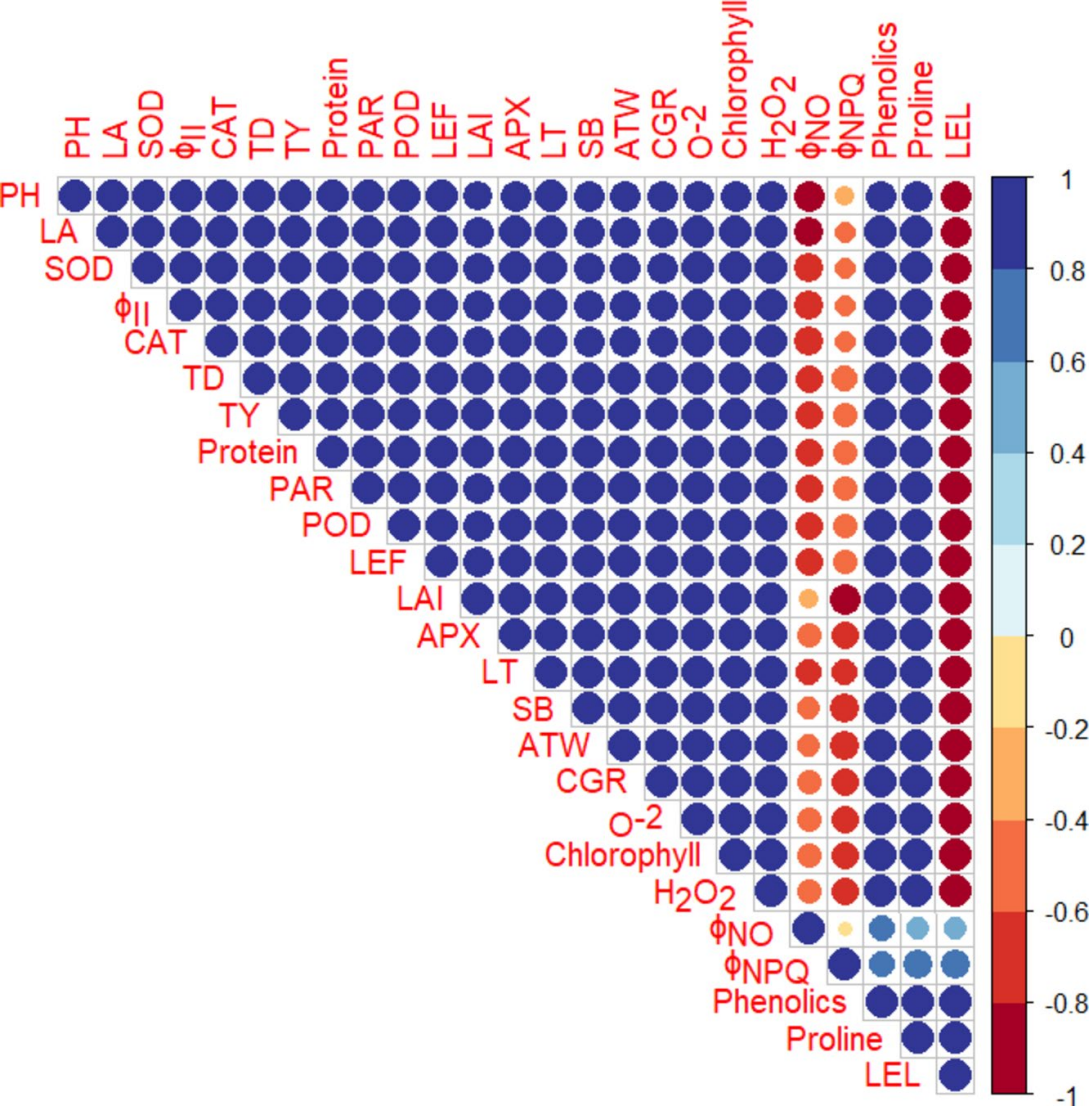
Pearson correlation (*P* ≤ 0.05) among the determined traits of potato cv. Sutlej; PH = plant height, LA = leaf angle, SOD = superoxide dismutase, Φ_II_ = quantum yield of photosystem II, CAT = catalase, TD = tuber diameter, TY = tuber yield; PAR = photosynthetically active radiation, POD = peroxidase, LEF = linear electron flow; LAI = leaf area index, APX = ascorbate peroxidase, LT = leaf thickness, SB = shoot biomass, ATW = average tuber weight, CGR = crop growth rate, O^–2^= superoxide anion, H_2_O_2_ = hydrogen peroxide content, Φ_NO_ = non-regulatory energy dissipation, Φ_NPQ_ = non-photochemical quenching, LEL = leaf electrolyte leakage.

**Fig. 6:**
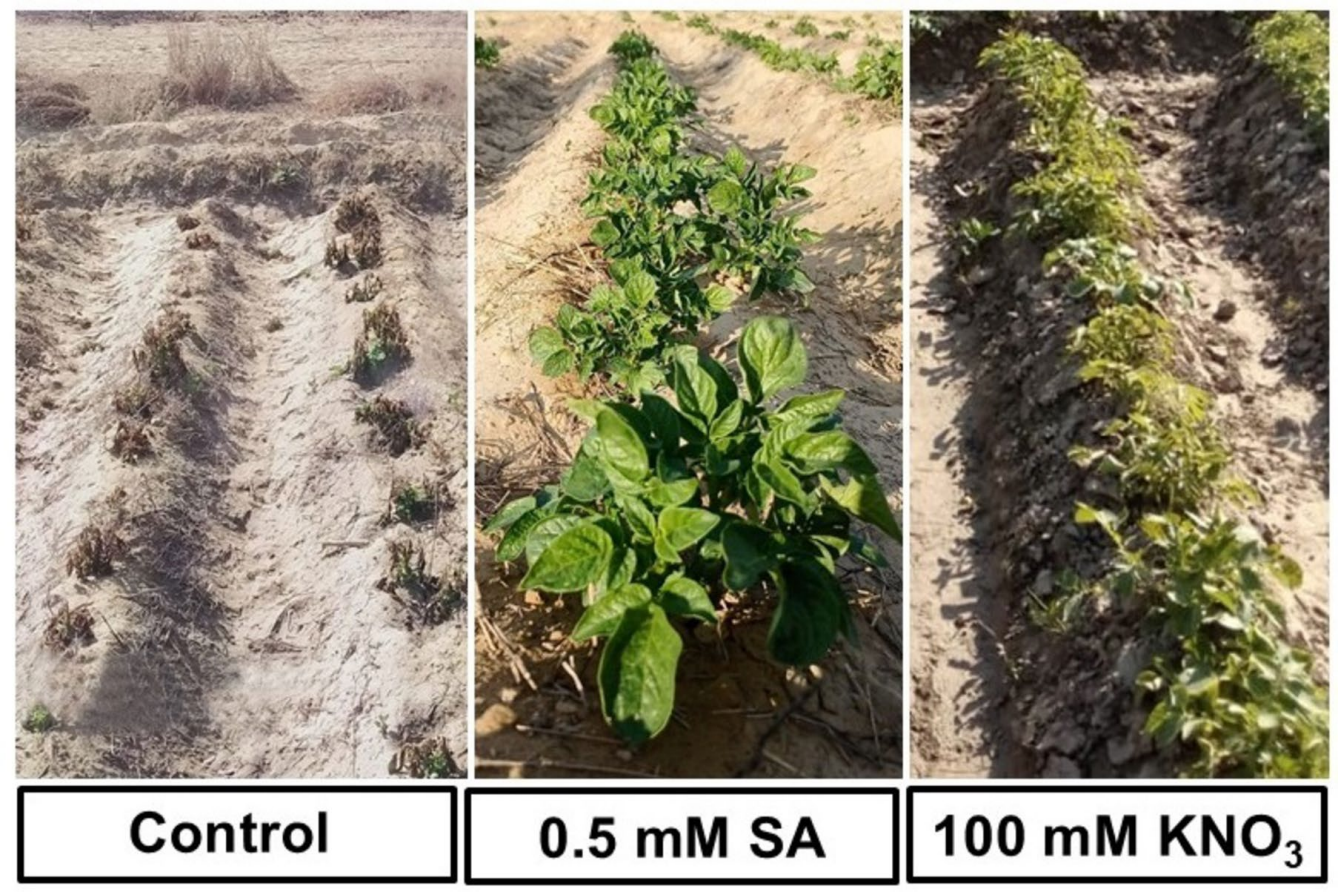
Appearance of potato plants under the effect of salicylic acid (SA), potassium nitrate (KNO_3_) and control after 20 days of frost occurrence.

The current study investigates the effects of foliar sprays of SA and KNO_3_ under the frost stress in comparison with the control. The foliar application of 0.5 mM SA and 100 mM KNO_3_ had a substantial effect on the morphological attributes including plant height, LAI, shoot biomass, CGR, average tuber weight, tuber diameter, and tuber yield compared to the control (Fig. 2A–D). The positive effect of SA in our study is supported by Souri and Tohidloo^36^, who found a significant improvement in the shoot biomass and leaf proline content with the foliar spray of SA compared to the control. Our findings are also identical to those of Faried et al.^37^, who found a significant effect of SA on growth, antioxidative enzyme activities, and osmolytes of potato plants under salt stress. In another study, a pronounced positive effect of SA on growth, yield, and antioxidative enzymes of grapes was noted by Jalili et al.^38^ under frost stress. The current study also proves the findings of Azmat et al.^39^, who observed that the foliar application of SA induced drought stress tolerance in wheat plants by enhancing shoot biomass, photosynthetic efficiency, osmolytes, and antioxidant defense system. Moreover, La et al.^40^ noticed a substantial amelioration in adverse effect of drought on various growth and yield attributes of *Brassica rapa* with the application of SA. The positive influence of KNO_3_ on plant growth and development is supported by the findings of Abeed et al.^41^, who reported an ameliorative effect of KNO_3_ on growth and yield of radish. Similarly, foliar application of K_2_SO_4_ improved frost stress tolerance in tomato^42^. All the studied morphological attributes were found to have a strong positive correlation (*r* ≥ 0.8) (*P* ≤ 0.05) with ΦII, chlorophyll content, PARs, leaf thickness, LEF, O^–2^ and H_2_O_2_ accumulation, antioxidative enzyme activities, proline and phenolic content, while a strong negative correlation (*r* ≤ − 0.8) (*P* ≤ 0.05) with Φ_NO_ and leaf electrolyte leakage (Fig. 5). So, the mechanism behind the improved growth and yield of potato, involves improved photosynthetic efficacy, striking PARs, electron transport chain, and resultantly increased chlorophyll content, triggered accumulation of reactive oxygen species (ROS) and hence generation of antioxidative enzyme activities and phenolics in response; enhanced production of osmolytes i.e., proline and decreased leaf electrolyte leakage; as a result, the plants overwhelmed the frost stress. Overall, the visual difference of the effect of the control and 0.5 mM SA and 100 mM KNO_3_ applications is evident from the appearance of potato plants after 25 days of frost occurrence (Fig. 6).

The positive influence of SA and KNO_3_ on chlorophyll fluorescence has a tremendous role in overcoming the oxidative stress^43^. The chlorophyll fluorescence is a measurement of the amount of light energy absorbed by chlorophyll molecules in plants and re-emitted as fluorescence light^44,45^. The current study also found that SA (0.5 mM) and KNO_3_ (1%) application raised the relative chlorophyll content of potato leaves by 14.5% and 8.9%, respectively (Fig. 3B). In this study, leaf thickness displayed a substantial positive correlation (*r* ≥ 0.8) (*P* ≤ 0.05) with subcellular CO_2_ and photosynthetic rate in the leaves of potato (Fig. 5). The thickness of leaves is beneficial for photosynthesis because chloroplasts, even those positioned deeper within the leaves, can absorb a sufficient amount of light^46^. Leaf angle was measured in this work because it has a substantial impact on chlorophyll fluorescence in plants by regulating light absorption, distribution, and physiological responses to varied light circumstances^47^. The leaves, which are perpendicular to incoming sunlight, receive the lightest, permitting chloroplasts to capture more photons and begin photosynthesis more effectively. On the contrary, plants at low angles receive lower levels of light, resulting to less chlorophyll stimulation and slower rates of photosynthesis^48^. Photosynthetically active radiation (PARs) is another key photosynthetic characteristic, representing the proportion of incoming light (400–700 nm) that can be used for photosynthesis. In the current study, leaf angle had a substantial positive relationship with PARs (Fig. 5), indicating that leaves inclined broader towards light coming in received more PARs, hence boosting photosynthesis. The results are consistent with those of Yirdaw and Luukkanen^49^, who measured the transmittance rate of PARs in five distinct plant species and discovered that leaf angle plays a critical role in light absorption for photosynthesis. Leaf thickness positively correlates with Φ_II_, indicating that seedlings with thicker leaves may have more chloroplasts per unit leaf area (Fig. 5). Numerous earlier investigations have demonstrated an increase in ΦII with a rise in leaf thickness^50–53^.

**Fig. 7:**
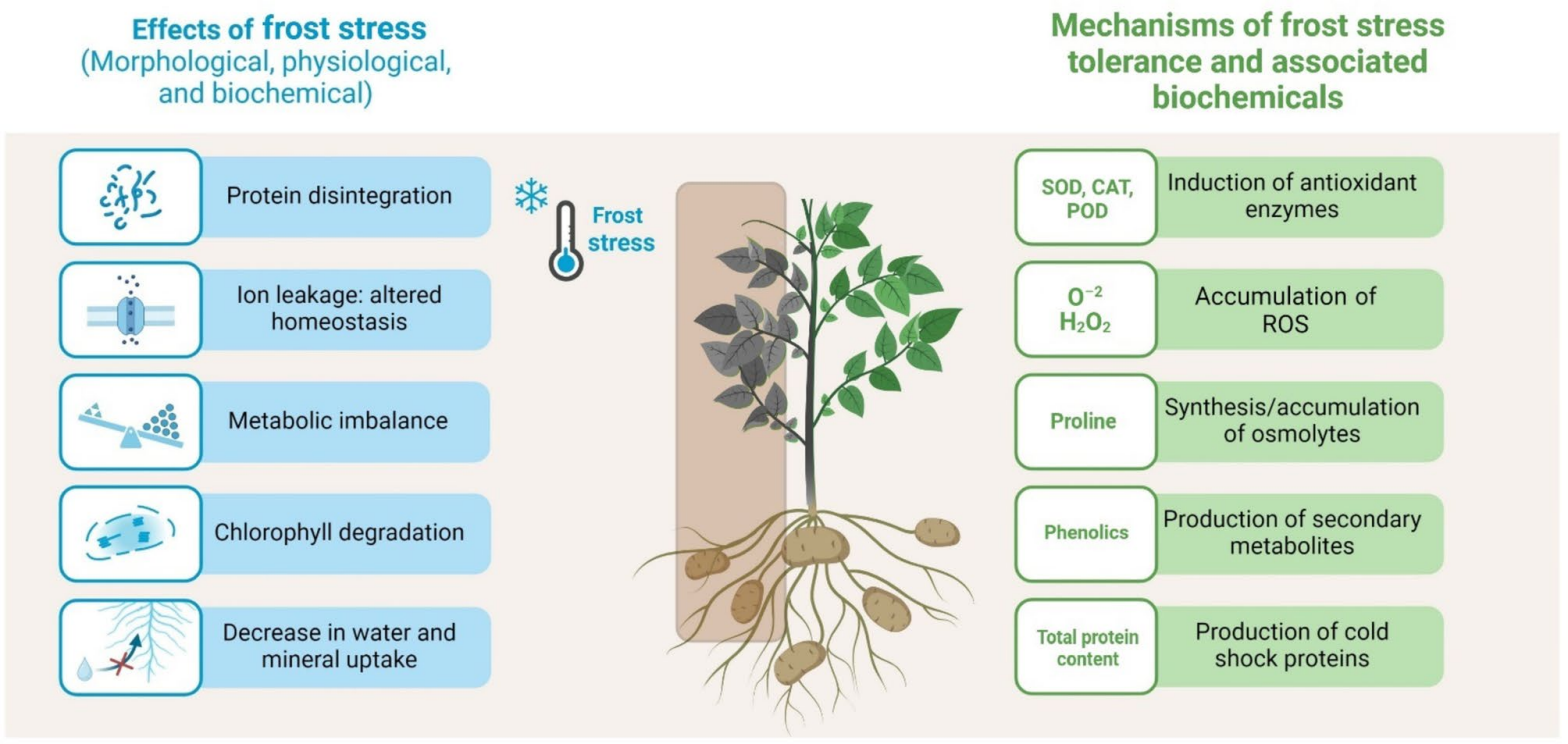
The proposed mechanism of frost stress and its tolerance in potato plant.

The earlier works indicated that SA and KNO_3_ are involved in abiotic stress signaling pathways, induction of abiotic stress tolerance and improved growth and yield^31,54–56^. Redox homeostasis in the plants have been achieved through application of SA and KNO_3_ via regulation of ROS production and their neutralization by activation of antioxidant enzymes e.g., SOD, CAT, POD, and APX as well as osmolytes including proline^37,57^. Likewise, in the current study SA and KNO_3_ assisted in maintaining redox balance that has been supported by the correlation between the studied variables (Fig. 5). A positive correlation (*r* ≥ 0.8) (*P* ≤ 0.05) of tuber yield was noted with CAT, SOD, POD, and APX (Fig. 5). Similarly, proline also had a positive relationship with SOD and total phenolic content (Fig. 5). Frost stress resulted in the production of ROS that resulted in cellular dysfunction and decrease in Chlorophyll content. SA and KNO_3_ may have played the role of an antioxidant that reduced O^–2^ and H_2_O_2_ concentrations in chloroplast and reduced the impact of frost stress on Chlorophyll content under frost stress (Fig. 5). Stress enhanced ROS production that induced oxidative stress, oxidized biomolecules including lipids, proteins, and nucleic acids, and disrupted redox equilibrium^58^. It was also noticed that the application of SA and KNO_3_ during frost stress first elicited medium stress by generating O^–2^ and H_2_O_2_ that in turn enhanced the generation of enzymatic antioxidants (e.g., SOD, CAT, POD, and APX). Furthermore, SA had a tendency to interact with antioxidant enzymes such as CAT, SOD, POD, and APX which are related to redox homeostasis, ROS metabolism and thereby, enhanced defense mechanism in plants. Apart from that, SOD enzyme changed O^–2^ into H_2_O_2_ that was reduced to water by CAT and various oxidases through Halliwell Asada pathway^59^. Likewise, the exogenous application of SA in tomato^60^ and KNO_3_ on radish^41^ increased the antioxidant enzyme activities such as SOD, CAT and POD. Frost stress reduced the levels of soluble protein. SA (0.5 mM) application increased total soluble protein content (Fig. 5D) by inducing protein-kinase synthesis, increasing nitrate reductase activity, and regulating metabolic pathways and cell division, differentiation, and morphogenesis through enhanced antioxidant activities, resulting in improved plant growth. Proline and total phenolic content increased under frost stress after foliar spray of SA (0.5 mM) in the leaves of potato plants. From the above results, it is clear that frost has a destructive effect on control plants but SA and KNO_3_ application has regulated the proline content in the cytosol, activated adaptive responses to stabilize membranes, scavenged ROS, and maintained proteins and enzymes which led to increased photosynthetic efficiency and plant productivity (Fig. 7).

## Conclusion

Foliar application of 0.5 mM salicylic acid has shown a promising antistress effect on field-grown potatoes cv. Sutlej by improving their defense mechanism compared to the 100 mM potassium nitrate and control. However, additional studies are required to test some more intervals to stabilize the rate of potassium nitrate for its maximal frost stress tolerance potential. .

## Materials and methods

### Experimental site

A field study was carried out at Horticulture Experimental Area (Longitude: 71°45’ 53.6” E; Latitude: 29°22’ 17. 4” N), Department of Horticultural Sciences, Faculty of Agriculture and Environment, The Islamia University of Bahawalpur, Pakistan during 2022–23 cropping season. This area is in the Cholistan desert and characterized by a subtropical climate. The minimum and maximum mean temperatures in this area are 10 °C and 40 °C, respectively. The mean annual rainfall varies between 100 and 200 mm. The minimum and maximum air temperature ranges for each month in the crop period (2022–23) were determined in the experimental period. The soil of Cholistan is described as saline, alkaline, and gypsiferous. The sub-surface water is slightly saline and has a soluble salt content of more than 900 mg L^–1^^1^. Prior to the trial, soil samples were collected from the experimental site and their physical and chemical properties were determined following the procedures outlined by Ryan et al.^61^. Five core soil samples in total from the experimental site were taken at two horizons: 0–15 cm and 16–30 cm using an auger. Thereafter, the soil obtained from five random cores was combined. The soil texture, electrical conductivity (EC), saturation and cation exchange capacity^1^, pH^62^, available phosphorus^63^ and potassium^64^, total nitrogen^65^ and organic matter^66^ were measured in both layers. Similarly, irrigated water was tested for physical and chemical qualities.

### Planting material

Potato cv. Sutlej with a dense and smooth greenish foliage, slightly erect, round to oval tuber shape, yellow skin and white flesh was used as planting material. Permissions or licenses were obtained to collect Potato cv. Sutlej, from Regional Agricultural Research Institute (RARI), Bahawalpur, Pakistan, before starting the research.

### Tested chemicals against frost

In the current experiment, two different chemicals were tested including salicylic acid (SA) (Analytical grade, Sigma Chemicals) and potassium nitrate (KNO_3_) (Analytical grade, Sigma Chemicals) against the frost stress. The levels of SA and KNO_3_ were optimized in a preliminary study using 0.5, 0.75, and 1.0 mM concentrations for SA, and 50, 100 and 150 mM concentrations for KNO_3_. The application of 0.5 mM SA and 100 mM KNO_3_ was carried out thrice in the cropping season at one month interval during the morning beginning from November 5, 2022. The knapsack sprayer was used for foliar application of tested chemicals. The control plants were sprayed with distilled water.

### Experimental protocol

The medium-sized, healthy seed tubers with average tuber weight 65 ± 5 g of cultivar Sutlej were planted manually at a P×P spacing of about 15 cm and 0.1 m depth in the ridges developed 75 cm apart on October 5, 2022 and tubers were harvested 135 days after sowing, on February 17, 2023, with the help of a spade.

### Field and laboratory measurements

The data relating to morphological, fluorescent, and biochemical indices of potato was assessed from five randomly selected plants.

### Morphological indices

The morphological indices like plant height, leaf area index, crop growth rate and shoot biomass were determined three weeks after the third (last) application of SA and KNO_3_ on January 25, 2023. The plant height was measured using a measuring tape. The leaf area index was estimated by dividing the leaf area per unit of land which was derived from the below equation as adopted by Haider et al.^1^.

1 $$\:Log10\:\left(leaf\:area\:in\:cm2\right)=2.06\times\:Log10\left(leaf\:length\:in\:cm\right)-0.458$$

The crop growth rate was determined from the samples uprooted on January 25, 2023 and thereafter one week on February 1, 2023. Then, fresh weight of these samples was recorded and air-dried by placing the samples on clean newspaper. After that, the samples were kept in a hot dry oven (SLN 115, Pol-Eko, Poland) at 70 °C until their dry weight was stabilized by multiple sample weighings. The dry weight values were then placed in the below equation in order to obtain the crop growth rate values. Where Wf represents dry weight of the final sample, Wi represents dry weight of the initial sample, whereas $\:{T}_{\text{f}}-{T}_{\text{i}}$ represents the time period.

2 $$\:Crop\:growth\:rate\:\left(CGR\right)=\frac{{W}_{\text{f}}-{W}_{\text{i}}}{{T}_{\text{f}}-{T}_{\text{i}}}\:\:\:\:\:\:\:\:\:\:\:\:\:\:\:\:\:\:$$

The average tuber weight, tuber diameter, and tuber yield were assessed after harvesting. The tuber weight was measured using an electronic scale (DM-01, ScaleTech, China). The tuber diameter was recorded using vernier caliper (IP67, BEAPO Hardware Industrial Company, China). The tuber yield per hectare was calculated from the following formula:

3 $$\:Tuber\:yield\:per\:hectare=\frac{Yield\:of\:treatment\--plot\:\:}{Area\:of\:treatment\--\:plot}\times\:Area\:of\:hectare$$

### Fluorescent indices

The fluorescent indices like leaf angle, quantum yield of photosystem II (Φ_II_), chlorophyll content, photosynthetically active radiations (PARs), leaf thickness, linear electron flow (LEF), non-photochemical quenching (Φ_NPQ_), and non-regulatory energy dissipation (Φ_NO_) were also recorded on January 25, 2023 with the help of a hand-held instrument namely MultispeQ-Beta connected with the PhotosynQ platform^67^.

### Biochemical indices

The concentration of O^− 2^ in the leaf tissues of potato was measured according to the procedure explained by Hasan et al.^68^. The H2O2 content were determined following the methodology of Haider et al.^69^. For the determination of antioxidative enzymes, the mature leaves of potato cv. Sutlej were crushed with the help of cold mortar and pestle and mixed with 2 ml of phosphate buffer (pH = 7). The mixture was then centrifuged at 10,000 rpm in Rotofix 46 centrifuge (Hettich, Kirchlengern, Germany) for 5 min at 4 °C. The concentration of antioxidative enzymes was assayed once the supernatant had been prepared. The determination of the activities of catalase (CAT) (EC 1.11.1.6), peroxidase (POD) (EC 1.11.1.7), ascorbate peroxidase (APX) (EC 1.11.1.11), and superoxide dismutase (SOD) (EC 1.15.1.1) were estimated according to the method described by Haider et al.^69^. The content of total soluble protein in potato leaves was also determined adopting the protocol of Haider et al.^69^. The proline content was estimated using the method of Bates et al.^70^. The total phenolic content was determined using the protocols earlier adopted by Haider et al.^71^. To determine the electrolyte leakage in the tissues of potato leaves, the excised leaf samples weighing 2 ± 0.1 g were submerged in deionized water (25 ml) and kept at ambient temperature (~ 27 °C). After recording the initial EC (ECi), the samples were boiled for 15 min at 100 °C and the final EC (ECf) was recorded. EL of each sample was then determined using the below formula:

4 $$\:EL\:\left(\text{\%}\right)=\frac{{EC}_{\text{i}}}{{EC}_{\text{f}}}\times\:100\:\:\:\:\:\:\:\:\:\:\:\:\:\:\:\:\:$$

### Data analysis

The study was designed following a randomized complete block design (RCBD) with four replicates. All the collected data was processed on Microsoft Excel 2016. The analysis and means comparison of the treatments were carried out through analysis of variance (ANOVA) and least significant difference (LSD) test at *p* ≤ 0.05 using Statistix 9 (Analytical Software, Tallahassee, FL, USA). The correlation analysis among the determined attributes was conducted using the “corrplot” package of R Studio^72^.

## Data Availability

All the data related to this work can be sourced from the corresponding authors.

## Declarations

### Permission

Permissions or licenses were obtained to collect Potato cv. Sutlej, from Regional Agricultural Research Institute (RARI), Bahawalpur, Pakistan, before starting the research.

### Statement on guidelines

All experimental studies and experimental materials involved in this research are in full compliance with relevant institutional, national and international guidelines and legislation posing a conflict or bias.
